# Editorial: Rising stars in veterinary regenerative medicine: 2022

**DOI:** 10.3389/fvets.2023.1331168

**Published:** 2023-11-30

**Authors:** Laura Barrachina, Janina Burk, Iris Gerner

**Affiliations:** ^1^Regenerative Medicine Institute (REMEDI), Biosciences, University of Galway, Galway, Ireland; ^2^Department of Biomedical Sciences, Institute of Physiology, Pathophysiology and Biophysics, University of Veterinary Medicine, Vienna, Austria; ^3^Veterinary Tissue Engineering and Regenerative Medicine Group, Equine Surgery Unit, Department for Companion Animals and Horses, University of Veterinary Medicine, Vienna, Austria

**Keywords:** regeneration, veterinary medicine, orthobiologics, mesenchymal stromal (stem) cell, platelets

It all began at a fast pace, with much hope and little fear: when the regenerative medicine field gained momentum, veterinary medicine was at the forefront of clinical applications for patients with naturally occurring disorders. We have learned that, indeed, there may not be much to fear when it comes to local autologous therapies with adult progenitor cells, which have since then largely proven to be safe. Many case studies were performed with promising results, indicating good efficacy and thereby promoting the tremendous hopes associated with regenerative therapies. Nevertheless, despite a growing research community with rapidly expanding output, progress has slowed down and the field has not yet managed to meet all the high expectations. The more progress the field made, the more questions arose, and we only slowly begin to unravel the complex modes of action of different biologics, their interaction with host cells and tissues, and how to stratify well-responding vs. non-responding patients.

The veterinary profession has an important role to play in the development of regenerative therapies, representing the link between basic science and human clinical applications, owed to the central role of animal experiments in translational research. In addition, animals are an integral part of our society and economy, so they are patients on their own rights in need of advanced treatments. Progress in the field is driven by both the need for companion animal treatments and the value of preclinical data gained from animal models and veterinary patients. Animal models require careful selection and design to ensure they are fit-for-purpose and provide optimal predictive validity while, at the same time, meet ethical animal welfare requirements. In spite of this, regenerative therapies for human patients are often tested in animals that do not mimic the human anatomy and pathophysiology. Small animals, specifically mouse and rat models, are valuable for research into mechanisms of disease and fundamental biology, but findings from these models rarely translate into human clinical applications. Beyond anatomical and physiological differences, the main reason is that experimentally mimicking the high complexity of multifactorial pathologies is extremely challenging. Large animals are well-accepted, well-established and clinically relevant models that commonly suffer from naturally occurring disease/injuries with similar pathophysiology to the human in terms of etiology and risk factors, including, among others, over-exercise, age and genetic factors. Therefore, developing the veterinary regenerative field is important to improve animal health and revert benefit to human medicine, while diminishing the need for experimental animals. However, while the complexity of orthobiologics requires in-depth research, the veterinary research community faces specific challenges associated to working with non-conventional species. Intensifying the efforts is warranted by the unique landscape offered by the veterinary regenerative field.

Moving the field of regenerative medicine forward requires the cooperation of basic researchers, human and veterinary medical scientists. A big share of the affiliated research is contributed by dedicated young researchers willing to advance the standard of animal care. With this article collection, our goal is to promote promising young scientists in the field of veterinary regenerative medicine. This Research Topic compiles current work of several groups and their young researchers. The collection offers representative insight into the current status of the field, with in-depth studies on long-existing topics, such as the sources and characterization of mesenchymal stromal cells and blood products (Heilen et al.; Melzer et al.; Miguel-Pastor et al.; Andrietti et al.; Phyo et al.) and the clinical application of orthobiologics in osteoarthritis (Mayet et al.). Several articles highlight the importance to understand the immunological properties of orthobiologics (Cequier et al.; Pezzanite et al.; Moellerberndt et al.). In addition, fields which emerged more recently, such as extracellular vesicles and induced pluripotent stem cells, as well as new areas of clinical applications, are discussed in review format (Adamič and Vengust; Barrachina et al.; Jammes et al.; Weeratunga et al.).

We expect that this Research Topic not only holds a great scientific potential highlighting the progress in the veterinary regenerative medicine field, but may also show that human and veterinary medicine share a lot of research interests. The demand for higher standards of care for animals and for better and more ethical translational models is leading to a rapid advancement of the veterinary regenerative field and to the emergence of new frontiers. Thus, an “army” of researchers will be needed to truly unravel and exploit the potential of the veterinary regenerative field. Regenerative medicine is an exciting area of research for young scientists with many different profiles and with a multitude of interests, as the field requires highly multidisciplinary teams, from cell biologists and clinicians to engineers and bioinformaticians. However, developing a career in veterinary regenerative medicine does not come without challenges. For those with clinical interest, it may be difficult to combine research and clinical work as both are highly demanding and time-consuming. Fundraising may also be more difficult than in other research areas as there are very few specific calls for veterinary research, and some may feel discouraged by the competition with human-centered research. Opportunities for dedicated training, dissemination or networking (e.g. conferences, meetings) are sometimes hard to find, as the biggest regenerative medicine events are focused on applications in humans. However, the veterinary community is gaining presence in some of these meetings and is creating new spaces for interaction, which is the key for promoting engagement. Finally, as in other fields, supporting supervisors and institutions are of utmost importance to attract and promote young researchers. Owed to the increasing relevance of veterinary regenerative medicine and the unique challenges that are faced, the rising stars between us should be supported by all possible means.

## Author contributions

LB: Writing—original draft, Writing—review & editing. JB: Writing—original draft, Writing—review & editing. IG: Writing—original draft, Writing—review & editing.

